# Maternal Apocynin During Experimental Preeclampsia Prevents BBB Permeability and Increased Vascular Tone of Cerebral Arteries in Male but Not Female Rat Adult Offspring

**DOI:** 10.1111/micc.70044

**Published:** 2025-12-17

**Authors:** Milena Esposito, Sarah M. Tremble, Marilyn J. Cipolla

**Affiliations:** ^1^ Department of Neurological Sciences University of Vermont Larner College of Medicine Burlington Vermont USA; ^2^ Department of Biology, Ecology and Earth Science University of Calabria, Arcavacata di Rende Calabria Italy; ^3^ Department of Obstetrics, Gynecology and Reproductive Sciences University of Vermont Larner College of Medicine Burlington Vermont USA; ^4^ Department of Pharmacology University of Vermont Larner College of Medicine Burlington Vermont USA

**Keywords:** blood–brain barrier, cerebral circulation, offspring, oxidative stress, preeclampsia

## Abstract

**Objective:**

We examined if exposure to experimental preeclampsia (ePE) impacts vascular permeability and reactivity of posterior cerebral arteries (PCAs) in adult offspring, and if maternal antioxidant treatment prevents these effects in adult male and female offspring.

**Methods:**

Offspring (F1) from rats with Normal pregnancy (NormPreg_F1), ePE_F1 and ePE treated with apocynin (ePE + apo_F1) were weighed at p24, p31, p38, and p45. Maternal apocynin was administered weekly in drinking water at a dose of ∼24 mg/kg (3 mM) on gestational days 11–20. Blood–brain barrier (BBB) permeability, structure and function of PCAs were measured from male and female adult offspring (*n* = 8/group, 12–29 weeks). Circulating inflammatory factors were measured by multiplex array.

**Results:**

Male ePE_F1 had smaller body weights than male NormPreg_F1 (*p* < 0.05) at all ages which was prevented by maternal apocynin. Female ePE_F1 body weights were less only at p24 which was prevented by maternal apocynin. PCAs from male ePE_F1 had increased BBB permeability versus male NormPreg_F1 which was prevented by apocynin (*p* < 0.05). These changes were not seen in female ePE_F1s. Maternal ePE did not affect PCA reactivity to inward potassium rectifier channel or voltage‐operated calcium channel activation, nor reactivity to L‐NAME and sodium nitroprusside. There were little differences in plasma inflammatory factors.

**Conclusions:**

ePE exposure had long‐term consequences on the cerebral circulation of adult male offspring. Understanding the underlying mechanism by which PE adversely impacts the brain of offspring may lead to the prevention of cognitive decline and stroke in adulthood.

**Abbreviations**
aCSF: artificial cerebral spinal fluid
Apo: apocynin
BBB: blood brain barrier
ePE: experimental preeclampsia
F1: offspring
HC: high cholesterol
ID: inner diameter
L‐NAME: N(G)‐nitro‐L‐arginine methyl ester
L_p_
: hydraulic conductivity
NOS: nitric oxide synthase
NVC: neurovascular coupling
PCA: posterior cerebral artery
PE: preeclampsia
ROS: reactive oxygen species
VOCC: voltage operated calcium channel

## 1 Introduction

Preeclampsia (PE) is a hypertensive disease exclusive to pregnancy, affecting 3–8% of pregnancies worldwide [1]. PE disrupts maternal and placental physiology through several mechanisms including immune system dysregulation, increased oxidative stress and aberrant remodeling of uterine spiral arteries which contribute to a hypoxic in‐utero environment [1]. Besides the sequalae on maternal health [2], the pathophysiology of PE profoundly impacts offspring health both prenatally and throughout life [3]. Offspring from women with PE—especially early onset PE—have a higher risk of cardiovascular diseases and cognitive decline [4]. Short‐term fetal complications include stillbirth, fetal growth restriction and prematurity [3]; long‐term consequences on offspring include increased risk of stroke, cardiovascular disease [5], and brain‐related dysfunction [6].

Offspring from PE pregnancies have a 4‐fold higher risk to develop impaired memory and cognition compared to offspring from healthy pregnancies [7]. In humans, impaired cognition in preeclamptic offspring is manifested as deficits in verbal [8] and arithmetic reasoning [9] and lower IQ [9] during childhood. In adulthood, there is evidence of decreased cognitive function and increased depressive symptoms [10]. Remarkably, male offspring are twice as likely to develop neurodevelopmental disorders including Attention Deficit Hyperactive Disorder and autism, compared to females [11]. This sexual dimorphic effect is possibly due to differences in genetics, hormones, and environmental factors during development [12, 13]. Studies conducted in offspring from animal models of PE have shown impaired spatial working memory and exploratory behavior, as well as reduced volume of cerebral and entorhinal cortex, and occipital lobe, suggesting impact on neurogenesis [14, 15]. Additionally, human offspring exposed to PE have an increased risk of developing hypertension [5], obesity [16], and stroke [17, 18], all of which are significant contributors of cognitive decline and worse cognitive performance [19, 20, 21].

Vascular cognitive impairment is the second most common cause of age‐related dementia [22]. The interplay between cerebral vessels and cognition involves sustaining an adequate blood flow and the integrity of the BBB to preserve the delicate brain milieu [23].

Local blood flow to brain regions, including the hippocampus, a deep brain structure central to memory and cognition [24], is regulated through a process known as neurovascular coupling (NVC) [25]. Decreased cerebral blood flow [26], disruption in NVC [27] and BBB breakdown [28] are associated with cognitive dysfunction. In animals models, maternal ePE induces irreversible and progressive changes in cerebrovascular function during prenatal life of offspring (embryonic day 19) [29] and in post‐natal life, which manifests as deficits in brain microvascular perfusion [30], reduced angiogenesis (post‐natal day 5) [31] and defective maturation and function of the middle cerebral artery (post‐natal day 30) [17, 32]. Although cerebrovascular impairment has been demonstrated as a result of exposure to PE, data addressing the posterior circulation, as well as the underlying mechanisms by which maternal PE promote brain dysfunction and cognitive decline in offspring, are still lacking.

Increased oxidative stress in the intrauterine environment may be a contributor of cognitive impairment in offspring, since it promotes neuronal degeneration [33], endothelial dysfunction [34], BBB disruption [35], and neuroinflammation. If in‐utero oxidative stress manifests during pregnancy, this could have detrimental consequences on the offspring brain including the vasculature. In the current study, we hypothesized that the adverse in‐utero environment, including high oxidative stress during ePE, causes long‐lasting dysfunction of PCAs. We studied the PCA because it perfuses the hippocampus, giving insight to the association between vascular health and cognitive function. To test this hypothesis, we used the antioxidant apocynin, a naturally occurring compound with broad antioxidant properties. Apocynin has been shown to attenuate oxidative stress, improve vascular function and protect against hypertension and fetal growth restriction in animal models of PE [36, 37]. Remarkably, apocynin is well tolerated, with no teratogenic effects reported, and has been associated with improved placental function and increased fetal weight [37]. We therefore compared offspring from ePE dams treated with the antioxidant apocynin during gestation vs. those without treatment or having healthy pregnancy. We measured vascular permeability, and changes in the structure and function of PCAs in adult offspring.

## 2 Methods

### 2.1 Statement of Ethics and Animals

All animal procedures were approved by the University of Vermont Institutional Animal Care and Use Committee and complied with the National Institutes of Health Guide for the Care and Use of Laboratory Animals. ARRIVE guidelines were followed. Female pregnant Sprague Dawley rats (12–14 weeks; 250–300g) were purchased from Charles River (Saint‐Constant, QB, Canada) and housed singly at the University of Vermont Animal Care Facility, an Association for Assessment and Accreditation of Laboratory Animal Care‐accredited facility. Animals had access to food and water ad libitum and maintained a 12‐hour light/dark cycle. Animal groups were randomized using an online randomization tool.

### 2.2 Model of Experimental Preeclampsia

Pregnant Sprague‐Dawley rats (*n* = 9) were either Normal Pregnant (NormPreg, *n* = 4) or ePE (*n* = 3). ePE was induced by feeding dams with a high cholesterol (HC) diet (Prolab 3000 rat chow with 2% cholesterol and 0.5% sodium cholate; Scotts Distributing Inc., Hudson, NH, USA) starting from embryonic day E7. The HC diet was continued during the lactation period, since the abrupt suspension of this diet may cause seizure. The model of ePE used has been previously shown to be associated with maternal dyslipidemia, endothelial dysfunction, systemic inflammation, increased blood pressure, decreased placental weight, and fetal growth restriction, all of which are common features of PE [38, 39]. One group of ePE rats (*n* = 2) was administered with apocynin (ePE + apo), an antioxidant (24 mg/kg body weight) in drinking water on days 11–22 of gestation. Apocynin treatment was suspended at the time of delivery. The dose of apocynin was based on previous study which demonstrated beneficial effects on maternal hippocampal arterioles after apocynin treatment [40].

### 2.3 Offspring

**FIGURE 1:**
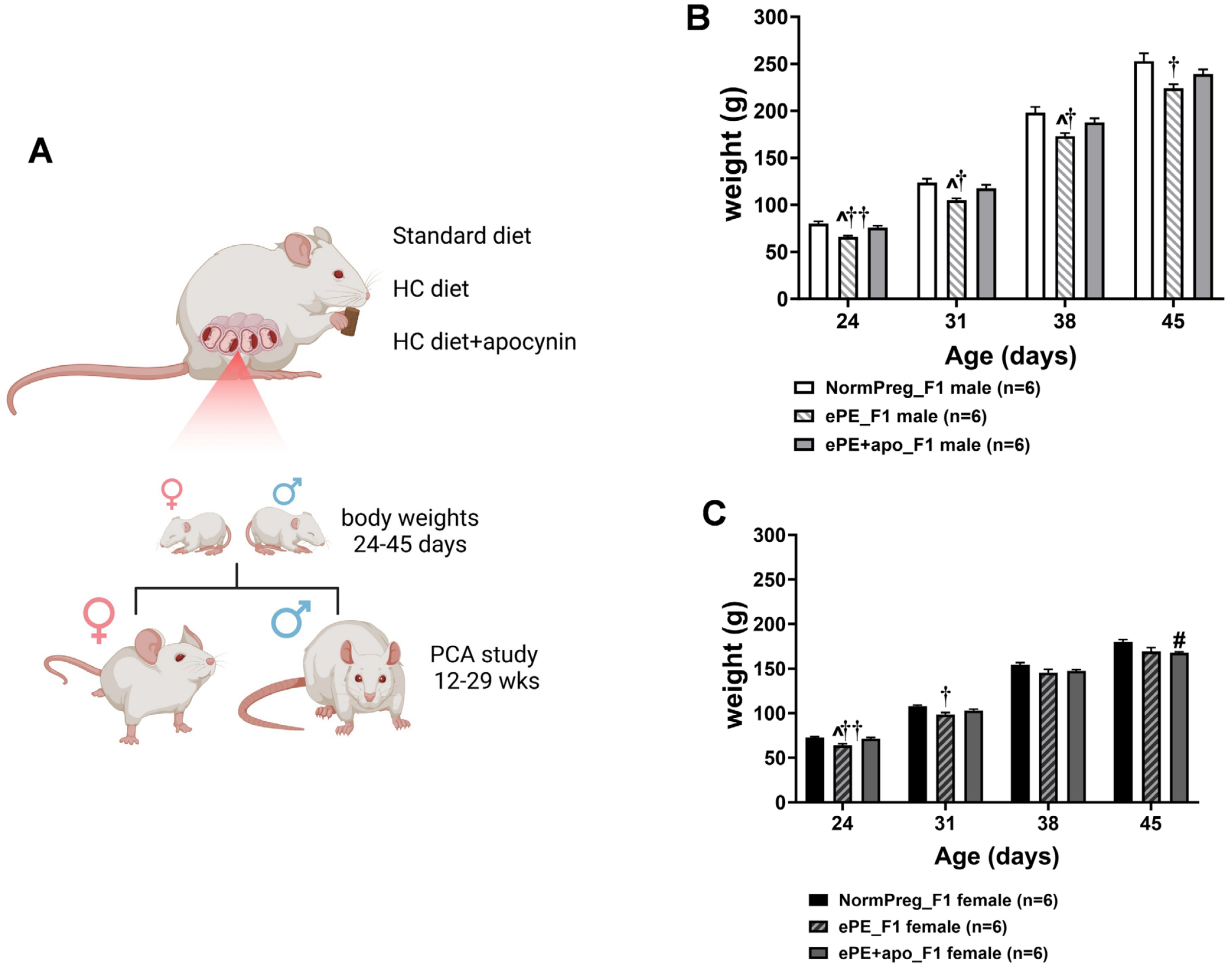
Study design and effects of maternal apocynin on offspring body weights in early life. (A) Study design. Dams were treated with either standard diet, HC diet or HC diet and apocynin in drinking water. Male and female offspring were weighed at p24, p31, p38, and p45, while PCAs function and structure were assessed between 12 and 29 weeks old. (B) Body weights of male offspring. ePE_F1 males had lower body weights at all ages versus NormPreg_F1 males (^††^ *p* < 0.01, ^†^ *p* < 0.05 represent pairwise differences in ePE_F1 vs. NormPreg_F1 by two‐way ANOVA Holm‐Šídák's post hoc). ePE + apo_F1 males had increased body weight at p24, p31, and p38 compared to male ePE_F1 (^*p* < 0.05 represent pairwise differences between ePE_F1 vs. ePE + apo_F1 by two‐way ANOVA Holm‐Šídák's post hoc). (C) Body weights of female offspring. Body weights were lower at p24 and p31 in ePE_F1 versus NormPreg_F1 females (^††^ *p* < 0.01, ^†^ *p* < 0.05 represent pairwise differences in ePE_F1 females versus NormPreg_F1 by two‐way ANOVA Holm‐Šídák's post hoc). Maternal apocynin treatment of females prevented the decreased body weight only at p24; ePE + apo_F1 females had increased body weight at p24 versus ePE_F1 (^*p* < 0.05 represents pairwise differences in ePE_F1 females vs. ePE + apo_F1 by two‐way ANOVA Holm‐Šídák's post hoc). However, this was not the case at subsequent ages (^#^ *p* < 0.05 represents pairwise difference between ePE + apo_F1 female vs. NormPreg_F1 by two‐way ANOVA Holm‐Šídák's post hoc).

All offspring were born in our facility, and at p21 male and female rats were group housed separately and fed regular chow and water until experimental use. Pups were randomly selected from two dams for each group. Two sets of animals were used for the study. The first set (*n* = 6/groups, 3 pups per sex per litter) was used for body weight and weighed at p24, p31, p38, and p45. The second set (*n* = 7–8/groups) was used for isolated vessel experiments at 12‐29 weeks old (adult). Figure 1A shows the design study. In the NormPreg_F1 group, one dam contributed 7 males and 5 females, the remaining 3 dams contributed 1 male and 2 female each. In the ePE_F1 group, one dam contributed 5 males and 6 females, the remaining 2 dams contributed 1 male and 1 female each. In the ePE + apo_F1 group, 1 dam contributed 5 females and 6 females, the other contributed 3 males and 2 females. The number of offspring per dam varied due to litter size and ethical considerations.

### 2.4 Inflammatory and Immune Response Markers in Offspring

Plasma was collected from trunk blood at the time of euthanasia into EDTA blood collection tubes (BD Vacutainer PPT, 362788, Franklin Lakes, NJ) and spun at 2500 *g* for 10 min and stored at −80°C until use. Samples were plated in duplicate on commercially available ProcartaPlex plates 2 or 7 plex plates (PPX‐02 and PPX‐07, ThermoFisherScientific, Waltham, MA) and read on the Luminex 200 (Luminex Corporation, Austin, TX) by the Laboratory for Clinical Biochemistry Research at the University of Vermont. VCAM‐1 and ICAM‐1 targets were run separately because plasma samples needed to be diluted 1:200 (PPX‐02). All remaining targets were run undiluted on one plate (PPX‐07).

### 2.5 Isolated Posterior Cerebral Artery (PCA) Protocol

After decapitation under deep isoflurane anesthesia (3% in oxygen), the brain was dissected and transferred to a petri dish filled with cold, aerated aCSF (artificial cerebrospinal fluid). Third order PCAs were dissected and sectioned into segments approximately 3–4 mm in length before mounting onto glass cannulas of a myograph chamber (Instrumentation and Model Facility at the University of Vermont, Burlington, VT, USA). Both cannulas and the chamber were filled with aCSF. The proximal end of the vessel was firmly attached to the glass microcannula using nylon threads, while the distal end was tied off and fixed to the distal cannula. Intravascular pressure was maintained by a pressure servo system (Living Systems Instrumentation, Burlington, VT, USA). Visualization of the vessel was achieved through a microscope connected to a TV camera and video dimension analyzer (Living Systems Instrumentation, Burlington, VT, USA). Continuous recording of the lumen diameter and wall thickness was facilitated by data acquisition software provided by WinDaq (Akron, OH). Detailed protocols for vessel cannulation, pressurization, and lumen diameter recording have been previously published [41]. Physiological conditions were maintained throughout the course of the experiment (pH 7.4 ± 0.2, 37.0°C ± 0.2°C) by a heat exchanger and aeration of aCSF.

### 2.6 Measurement of Hydraulic Conductivity (L_p_) in Isolated PCAs

After 1 h equilibration at 20 mmHg, the intravascular pressure was increased to 80.0 ± 0.5 mmHg, the automatic pressure controller was disengaged and the drop in pressure due to transvascular filtration across the vascular wall in response to hydrostatic pressure was measured every 5 min over a period of 40 min. The decrease in intravascular pressure per minute (mmHg/min) was converted to volume flux across the vessel wall (cm^3^) using a conversion curve, as previously described [42]. L_p_ was then determined by normalizing flux to surface area of the PCA (see Supporting Information for data calculations).

### 2.7 Reactivity Studies in Isolated PCAs

After 1 h equilibration and BBB permeability assessment, the PCAs were equilibrated for 10 min at 40 mmHg in aCSF, followed by 20 mmHg stepwise increases in pressure to 140 mmHg and the inner lumen diameter (ID) was recorded at each pressure once stable, to assess myogenic reactivity. The intravascular pressure was set to 80 mmHg for the remainder of the experiment. PCAs reactivity to pharmacologic agents was measured. The reactivity to increasing concentrations of extracellular potassium chloride (KCl, 3–50 mM) was performed. These concentrations of K^+^ are known to activate the inward rectifying potassium (K_ir_) channel and voltage operated calcium channels (VOCC), causing dilation and constriction, respectively. Following a wash‐out of KCl, a single‐concentration of nitric oxide synthase (NOS) inhibitor Nω‐Nitro‐L‐arginine methyl ester hydrochloride (L‐NAME, 10^−3^M) was added to the bath and the inner diameter was recorded once stable. In the presence of L‐NAME, a concentration response to sodium nitroprusside (SNP, 10^−8^–10^−5^ M), a donor of NO was performed, and diameters were measured at each concentration. At the end of each experiment, aCSF was replaced with aCSF containing zero calcium, and papaverine (10^−4^ M) and diltiazem (10^−5^ M) were added to fully relax the vessel. Passive structural measurements were made within the range pressure of 5–200 mmHg (see Supporting Information for data calculations).

### 2.8 Drugs and Solutions

Apocynin‐treated drinking water was prepared weekly by dissolving apocynin in hot tap water (60°C) with continuous mixing. The solution was allowed to cool to room temperature before being provided to the rats. aCSF was prepared weekly and stored at 4°C until use. Dextrose was added on the day of the experiment at a concentration of 5.5 mM. aCSF contained (mM): NaCl 122.0, NaHCO_3_ 26.0, NaH3PO_4_ 1.25, KCl 3.0, MgCl_2_ 1.0, and CaCl_2_ 2.0.

In the aCSF with higher concentrations of KCl (5, 10, 15, 20, 30, 40, 50 mM), the amount of NaCl was reduced in order to maintain constant osmolality. Zero calcium aCSF was prepared similarly to aCSF, substituting CaCl_2_ with 0.5 mM EGTA. aCSF was aerated with 5% CO_2_, 10% O_2_, and 85% N_2_ to maintain pH at 7.4 ± 0.2 throughout the experiment. Stock solutions of L‐NAME, SNP, papaverine and diltiazem were prepared weekly and stored at 4°C until use.

### 2.9 Statistical Analysis

Analyses were performed using GraphPad Prism 10.4.1. Normality was assessed via Shapiro–Wilk tests. Data were analyzed using two‐way repeated analysis of variance (ANOVA), two‐way ANOVA with Holm‐Šídák's or Tukey's correction for multiple comparisons, or mixed‐effects model, and a two‐tailed Welch's *t*‐test as needed. Data not normally distributed were analyzed by multiple nonparametric Mann–Whitney tests with Holm‐Šídák's correction for multiple comparisons or a single two‐tailed Mann–Whitney test as needed. All data are expressed as mean ± SEM; statistical significance was set at *p* < 0.05.

### 2.10 Exclusions

One female was excluded from the NormPreg_F1 cohort due to vessel damage. One male was excluded from the ePE_F1 cohort in the functional studies because it was a statistical outlier, identified using the ROUT method (Q = 1%) in GraphPad.

## 3 Results

### 3.1 Maternal ePE Caused Reduction in Body Weights in Male but Not Female Offspring That Was Prevented by Apocynin

To investigate whether exposure to ePE impacted offspring development, and whether maternal treatment with apocynin could prevent this effect, we measured pup weights at p24, p31, p38, and p45. A two‐way repeated measures ANOVA was conducted to assess body weight trajectories over time in NormPreg_F1, ePE_F1, and ePE + apo_F1 groups of offspring. Figure 1B shows body weights over time for male offspring. There were significant main effects of time (*F*(1.19, 17.82) = 3483, *p* < 0.0001) and group (*F*(2, 15) = 7.55, *p* = 0.0054), as well as a significant time × group interaction (*F*(6, 45) = 2.50, *p* = 0.036). These results indicate that body weight increased markedly across time points in all groups, but the rate and magnitude of growth differed among groups. Specifically, male offspring from the ePE_F1 group exhibited lower body weights compared to NormPreg_F1 controls, while maternal apocynin treatment partially normalized this deficit. Figure 1C shows body weights over time for female offspring. A two‐way repeated measures ANOVA was used to evaluate body weight trajectories over time in female offspring and showed a similar significant main effects of time (*F*(1.44, 21.65) = 3878, *p* < 0.0001) and group (*F*(2, 15) = 5.27, *p* = 0.0184), as well as a significant time × group interaction (*F*(6, 45) = 3.67, *p* = 0.0047). These results indicate that body weights of female offspring increased significantly over time in all groups but that both the magnitude and pattern of growth differed among groups. Specifically, female offspring from the ePE group exhibited slower or reduced weight gain compared to NormPreg_F1 controls, while maternal apocynin treatment (ePE + apo_F1) appeared to partially restore normal growth patterns. When considered together, these findings indicate that offspring growth was strongly influenced by maternal preeclampsia, with both sexes showing altered developmental trajectories that were partially rescued by maternal antioxidant treatment. Although both male and female offspring exhibited significant interaction effects, the growth pattern divergence was more pronounced in females, suggesting that female offspring may be more sensitive to the lasting developmental effects of maternal vascular dysfunction.

### 3.2 Maternal ePE Increased BBB Permeability in Male but Not Female Adult Offspring That Was Prevented by Apocynin

**FIGURE 2:**
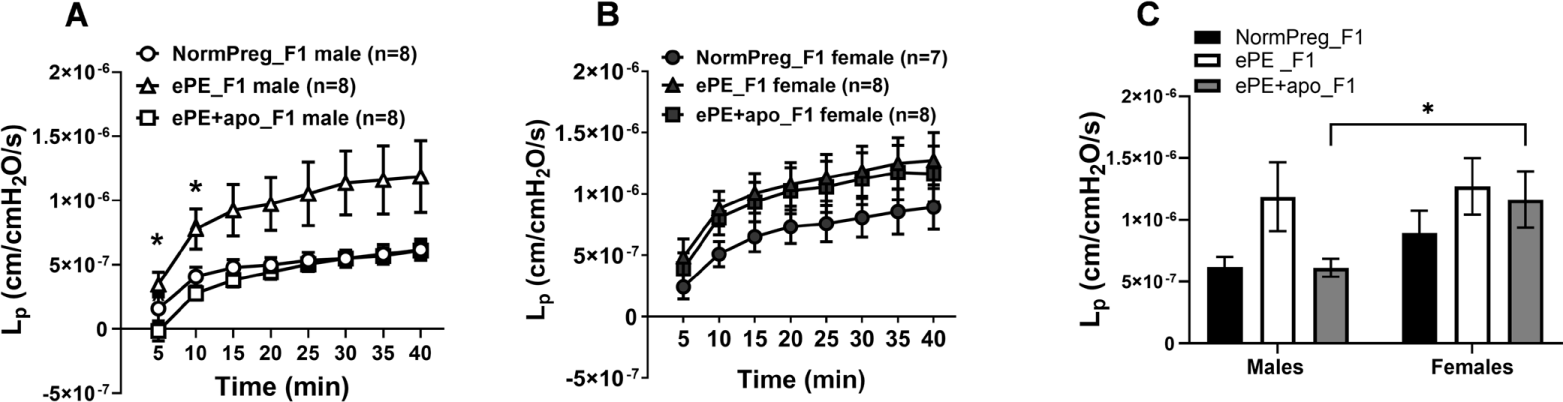
The effect of sex and maternal apocynin treatment on L_p_ over time of PCAs. (A) Graph showing L_p_ over 40 min of measurement in male offspring. L_p_ versus time was increased in PCAs from ePE_F1 versus NormPreg_F1 males that was prevented by apocynin. Two‐way repeated measures ANOVA revealed significant effects of time (*p* < 0.0001) and group (*p* = 0.0126), but no significant time × group interaction (*p* = 0.38). Asterisks indicate significant pairwise group differences at individual time points by Tukey's post hoc test (*p < 0.05 ePE_F1 vs. ePE + apo_F1). (B) L_p_ over time in female offspring. There were no statistical differences in L_p_ between ePE_F1, NormPreg_F1, and ePE + apo_F1 females by two‐way repeated measured ANOVA. (C) Graph showing L_p_ at 40 min comparing sex differences between the groups. A two‐way ANOVA was used to compare vascular permeability (L_p_) at 40 min between male and female offspring from all groups. The interaction between sex and group was not significant (*F*(2, 41) = 0.74, *p* = 0.48), indicating that the effect of group on permeability was similar in males and females. The main effect of sex showed a trend toward higher permeability in females (*F*(1, 41) = 3.64, *p* = 0.064), with mean L_p_ values of 1.12 × 10^−6^ ±1.24 × 10^−7^ versus 8.05 × 10^−7^ ± 1.08 × 10^‒7^ cm/cmH_2_O/s for females and males, respectively. The main effect of group also approached significance (*F*(2, 41) = 3.13, *p* = 0.054), suggesting modest differences among experimental groups that did not reach the threshold for statistical significance. However, pairwise comparison using Tukey's test for multiple comparisons found ePE + apo females were more permeable than their male counterparts (*p < 0.05 versus ePE + apoF1 males). Together, these results suggest that female offspring tended to exhibit slightly higher vascular permeability than males, but sex‐ and group‐related effects were not statistically significant at 40 min. Both sexes demonstrated similar patterns of response across experimental groups.

To explore underlying mechanisms by which ePE exposure may affect cerebrovascular function, we measured BBB permeability in PCAs. A two‐way repeated measures ANOVA was conducted to assess pressure‐dependent changes in vascular permeability (L_p_) from male F1 offspring from all groups. Figure 2A shows that there was a significant main effect of pressure (*F*(1.06, 22.27) = 28.78, *p* < 0.0001), indicating that permeability increased over time, as expected. A significant main effect of group (*F*(2, 21) = 5.42, *p* = 0.0126) demonstrated that overall L_p_ differed among the three groups. A post hoc test for multiple comparisons revealed that the pressure × group interaction was not significant (*F*(2.12, 22.27) = 1.03, *p* = 0.38), indicating that all groups exhibited a similar pattern of pressure‐dependent permeability changes. A significant subject effect (*F*(21, 147) = 27.3, *p* < 0.0001) reflected normal inter‐animal variability. Thus, exposure to experimental preeclampsia increased vascular permeability in male offspring, while maternal apocynin treatment partially normalized this effect, without altering the overall pressure–response relationship. BBB permeability was similar between NormPreg_F1, ePE_F1, and ePE + apo_F1 females (Figure 2B). A two‐way ANOVA was used to compare vascular permeability (L_p_) at 40 min between male and female offspring from all groups. The interaction between sex and group was not significant (*F*(2, 41) = 0.74, *p* = 0.48), indicating that the effect of group on permeability was similar in males and females (Figure 2C). The main effect of sex showed a trend toward higher permeability in females (*F*(1, 41) = 3.64, *p* = 0.064), with mean L_p_ values of 1.11 × 10^−6^ ± 1.24 × 10^−7^  versus 8.05 × 10^−7^ ±  1.08 × 10^−7^cm/cmH_2_O/s for females and males, respectively. The main effect of group just reached significance (*F*(2, 41) = 3.13, *p* = 0.05), suggesting modest differences among experimental groups that just reached the threshold for statistical significance. Together, these results suggest that female offspring tended to exhibit slightly higher vascular permeability than males, but sex‐ and group‐related effects were not statistically significant at 40 min. Similar findings were observed in flux and transvascular filtration measurements; see Supporting Information (Figures S1, S2).

### 3.3 Maternal ePE Caused Vascular Remodeling of PCAs in Male Adult Offspring That Was Prevented by Apocynin Treatment

**FIGURE 3:**
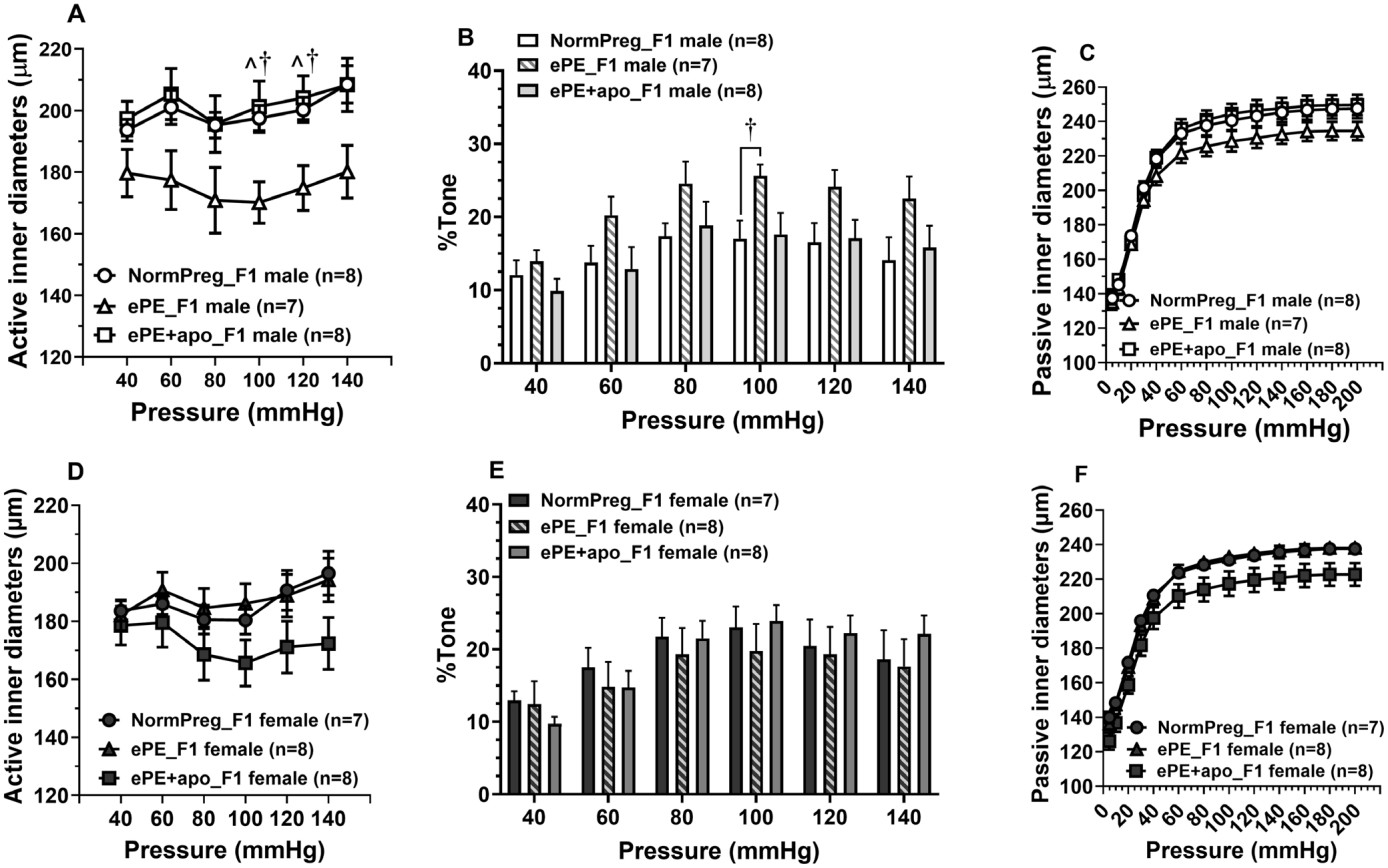
Effect of maternal apocynin treatment on offspring PCA active and passive lumen diameters. (A–C) Graphs comparing PCA active diameters, percent tone, and passive diameters from male offspring. PCAs from ePE_F1 versus NormPreg_F1 males had smaller active diameters (A) at 100 mmHg and 120 mmHg (^†^ *p* < 0.05 vs. NormPreg_F1 by two‐way ANOVA Holm‐Šídák's post hoc). Maternal apocynin treatment prevented this effect and male ePE + apo_F1 had larger lumen diameter versus ePE_F1 males at 100 and 120 mmHg (^*p* < 0.05 vs. ePE + apo_F1 by two‐way ANOVA Holm‐Šídák's post hoc). Myogenic tone (B) was increased in PCAs from ePE_F1 males vs. NormPreg_F1 males at 100 mmHg (^†^ *p* < 0.05 vs. NormPreg_F1 by two‐way ANOVA Holm‐Šídák's post hoc). Maternal apocynin treatment prevented the increase in tone. Lumen diameters of fully relaxed PCAs across the intravascular pressure 5‐200 mmHg (C) were not statistical different between ePE_F1, NormPreg_F1, and ePE + apo_F1. (D–F) Graphs comparing PCA active diameters, percent tone and passive diameters from female offspring. Active and passive lumen diameters were similar between female offspring. Percent of myogenic tone was not different between the female offspring.

To evaluate cerebrovascular structure and function in offspring exposed to ePE, pressure‐diameter relationships and vascular tone were assessed in PCAs from F1 males and females (Figure 3A–F). In male offspring (Figure 3A), a significant main effect of pressure (*F*(2.144, 42.98) = 3.795, *p* = 0.0278) and group (*F*(2, 20) = 5.134, *p* = 0.0159) were observed, indicating that active inner diameter changed significantly while increasing pressures and differed among experimental groups. The pressure × group interaction was not significant (*F*(10, 100) = 0.7898, *p* = 0.7898), suggesting a similar pressure–response pattern across groups. Post hoc analysis revealed that ePE_F1 males had significantly smaller diameters at 100 and 120 mmHg compared to NormPreg_F1 males (*p* = 0.0175 and *p* = 0.0384, respectively), while ePE + apo_F1 males showed significantly larger diameters than ePE_F1 males at the same pressures (*p* = 0.0248 and *p* = 0.0384), indicating partial normalization with maternal apocynin treatment. Analysis of vascular tone (Figure 3B) showed a significant main effect of pressure (*F*(2.08, 41.51) = 12.09, *p* < 0.0001), demonstrating pressure‐dependent changes in tone. The group effect approached significance (*F*(2, 20) = 3.15, *p* = 0.0647), suggesting a trend toward group differences, whereas the pressure × group interaction was not significant (*F*(4.15, 41.51) = 0.67, *p* = 0.6235). Post hoc comparisons revealed that ePE_F1 males exhibited significantly greater tone at 100 mmHg compared with NormPreg_F1 males (*p* < 0.01), and a trend toward higher tone relative to ePE + apo_F1 males (*p* = 0.064). In passive conditions (Figure 3C), there was a significant main effect of pressure (*F*(1.421, 28.42) = 1546, *p* < 0.0001), indicating that passive inner diameter changed markedly with pressure. Although no significant group effect was detected (*F*(2, 20) = 1.531, *p* = 0.2405), the pressure × group interaction was significant (*F*(24, 240) = 3.092, *p* < 0.0001), suggesting distinct pressure–diameter relationships among groups. Structurally, PCAs were relatively unchanged in cross‐sectional area, percent of distensibility and arterial stiffness within male ePE_F1 versus NormPreg_F1 versus ePE + apo_F1 (Figure S3A–C).

In female offspring, active diameter measurements (Figure 3D) revealed a significant main effect of pressure (*F*(2.43, 48.57) = 5.842, *p* = 0.0032), but no significant group effect (*F*(2, 20) = 1.680, *p* = 0.2116). A significant pressure × group interaction (*F*(10, 100) = 2.220, *p* = 0.0223) indicated that pressure–diameter responses differed among groups. For vascular tone in females (Figure 3E), a significant main effect of pressure was found (*F*(2.438, 48.76) = 27.07, *p* < 0.0001), while neither the group effect (*F*(2, 20) = 0.1529, *p* = 0.859) nor the pressure × group interaction (*F*(10, 100) = 1.517, *p* = 0.1442) was significant, indicating comparable pressure‐dependent tone responses across all groups. Finally, in passive diameters for females (Figure 3F), a significant main effect of pressure was observed (*F*(2.101, 42.02) = 1795, *p* < 0.0001), and the group effect approached significance (*F*(2, 20) = 3.288, *p* = 0.0582), suggesting a trend toward differences in overall diameter among groups. The pressure × group interaction was not significant (*F*(24, 240) = 0.8185, *p* = 0.7118), indicating that the pressure‐dependent diameter changes were similar across groups.

**FIGURE 4:**
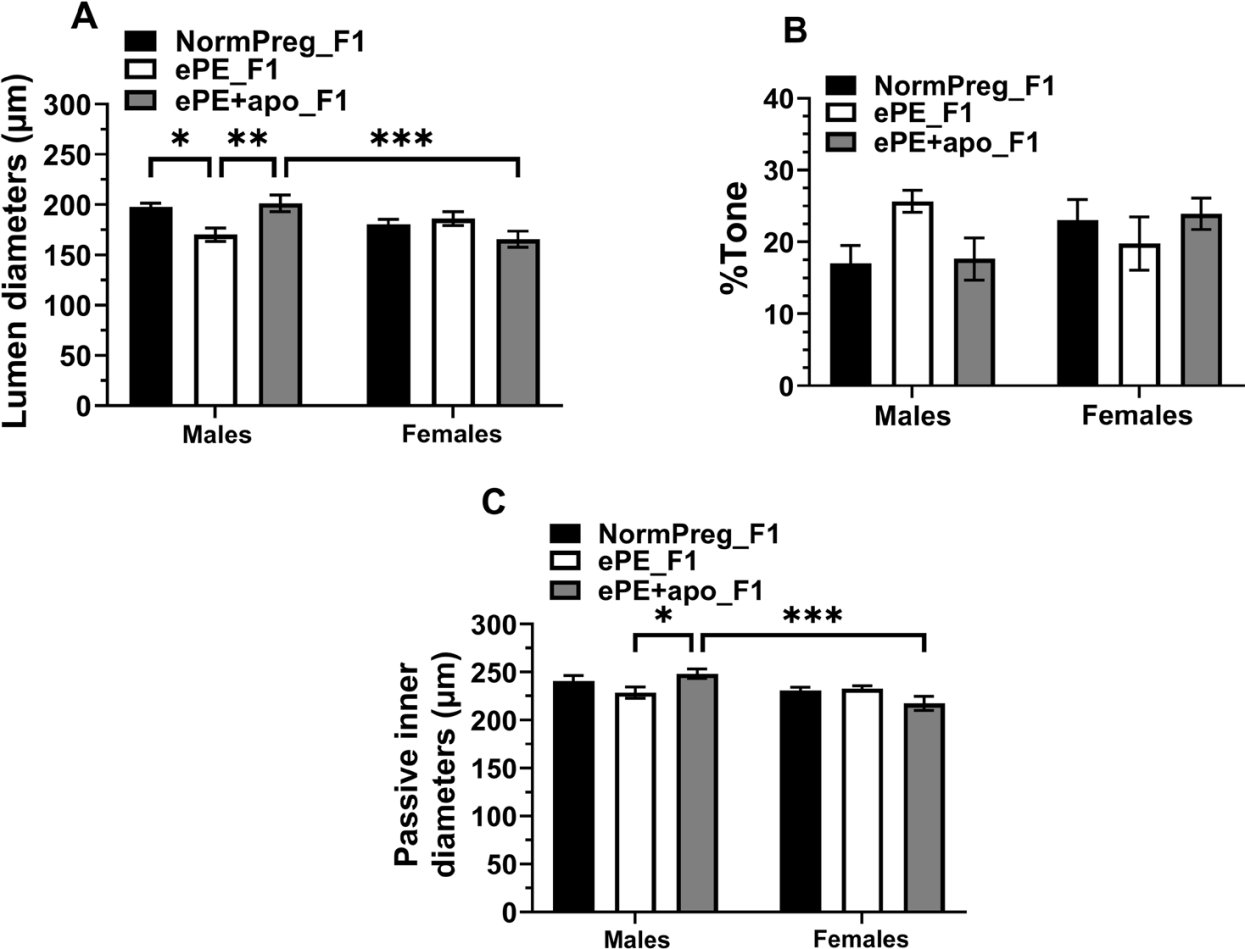
Effect of ePE and sex on PCA active diameters and tone. (A–C) Pairwise comparison showing active lumen diameters, % tone, and passive lumen diameters at 100 mmHg for all groups of male and female offspring, respectively. Between group comparison by two‐way ANOVA with posthoc Tukey's test revealed active diameter of PCAs from ePE_F1 males were smaller than NormPreg_F1 males that was prevented by apocynin; there were no differences between male and female ePE_F1. However, ePE + apo_F1 males had significantly larger PCAs than ePE + apo_F1 females both actively and passively, demonstrating a sex‐specific effect of maternal apocynin treatment on offspring PCAs. *p < 0.05; **p < 0.01; ***p < 0.001 as shown. There were no sex‐specific differences between male and female offspring in percent tone at 100 mmHg.

When we looked for sex‐specific differences by two‐way ANOVA at the physiological pressure of 100 mmHg, only ePE + apo_F1 groups were significantly different between males and females in active and passive diameters, but not percent tone (Figure 4). As noted above, ePE_F1 males had smaller active lumen diameters at 100 mmHg than NormPreg_F1 and ePE + apo_F1 males, suggesting maternal apocynin treatment prevented the smaller lumen diameters in males. The male‐specific effect of apocynin was confirmed with a significant pair‐wise difference between male and female ePE + apo_F1 groups. Similarly, no sex‐differences were found in passive inner diameters and cross‐sectional area for both NormPreg_F1 (Figure S4A,D) and ePE_F1 (Figure S4B,E). However, PCAs from ePE + apo_F1 males were larger and showed increased cross‐sectional area compared to ePE + apo_F1 females (*p* < 0.05, Figure S4C,F). Only NormPreg_F1 males had increased distensibility versus females (*p* < 0.05, Figure S5D). There was no sex differences found in the other groups (Figure S5E,F). Thus, maternal ePE induced vascular remodeling of PCAs, abolishing the sex‐specific difference observed in male and female NormPreg_F1. Maternal antioxidant treatment partially prevented the structural remodeling observed in ePE_F1 males.

### 3.4 Maternal ePE Had Little Effect on PCA Reactivity of Offspring

**FIGURE 5:**
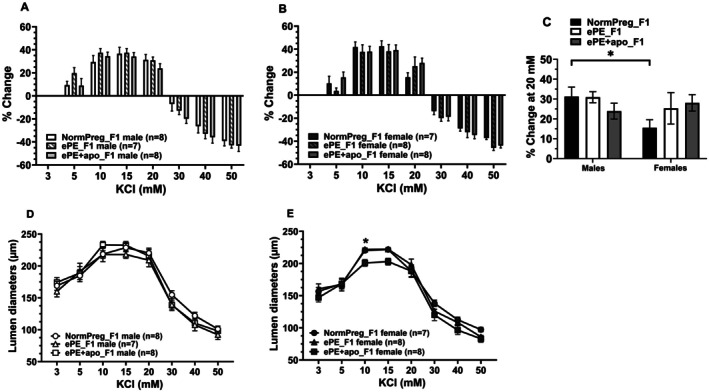
Effect of ePE and sex on PCA reactivity to increasing concentration of extracellular K^+^. Percent change in lumen diameter from baseline (3 mM) in response to increasing concentrations of K^+^ in PCAs from male (A) and female (B) NormPreg_F1, ePE_F1 and ePE + apo_F1. PCAs from all groups dilated to [K^+^] from 5 to 15 mM, then constricted from 20 to 50 mM. There were no differences in % dilation between groups. (C) Pairwise comparison between male and female offspring to dilation to 20 mM K^+^. NormPreg_F1 females dilated less than NormPreg_F1 males (*p < 0.05 vs. male by two‐way ANOVA with post hoc Tukey's for multiple comparisons). (D, E) Lumen diameter responses to increasing concentrations of K^+^. PCAs from all groups of animals dilated to K^+^ at concentrations < 15 mM, after which they constricted. Only PCAs from ePE + apo_F1 females had smaller diameters than the other female groups (*p < 0.05 by two‐way ANOVA with post hoc Tukey's for multiple comparisons); there were no other differences in diameters in response to K^+^.

To further examine whether exposure to ePE leads to dysfunction of PCAs into adulthood, vasoreactivity of PCAs to nitric oxide and K^+^, mediators involved in NVC, were analyzed. K^+^ has a bimodal effect, causing vasodilation at concentrations up to 15 mmol/L K^+^ and vasoconstriction at concentrations > 15 mmol/L, due to the activation of inward potassium rectifier channels and voltage‐operated calcium channels, respectively. Figure 5A,B show the % change in response to increasing concentrations of K^+^ in male and female groups, respectively; Figure 5D,E show the diameter responses. A two‐way ANOVA was performed to assess whether the percent change in vascular response to 20 mM KCl differed by sex (male vs. female) or maternal group (NormPreg_F1, ePE_F1, ePE + apo_F1). There were no significant main effects of sex (*F*(1, 40) = 1.96, *p* = 0.17) or group (*F*(2, 40) = 0.43, *p* = 0.65), and no significant sex × group interaction (*F*(2, 40) = 2.00, *p* = 0.15). However, a post hoc analysis for multiple comparisons revealed a significant difference between male and female NormPreg_F1 groups, suggesting a pairwise effect of sex that was present only in NormPreg offspring (Figure 5C). PCAs from all groups constricted to NOS inhibition with L‐NAME (Figure S6) and dilated to sodium nitroprusside (Figure S7A,B). Further, the EC_50_ for SNP was calculated for the groups (Figure S7C). These responses were similar between groups for both male and female offspring, that is, there were no sex or treatment differences.

### 3.5 Effect of Apocynin and ePE on Inflammatory and Immune Response Markers in Offspring

To assess whether ePE in dams caused systemic inflammation in adult offspring that might contribute to vascular dysfunction, we measured plasma inflammatory markers using multiplex array (Table 1). The LUMINEX analysis did not show difference in the levels of ICAM‐1, VCAM‐1, TNF‐ α, IL‐17A, IL‐1α, and IFNγ between ePE_F1, NormPreg_F1, ePE + apo_F1. However, ePE + apo_F1 females had increased levels of IL‐1β compared to ePE_F1 females (*p* < 0.01). Sex differences were found only in the levels of ICAM‐1 that was higher in ePE + apo_F1 males versus female (*p* < 0.05). Thus, there were little sex and treatment differences between groups.

**TABLE 1:** Inflammatory and immune‐response markers from adult offspring.

		NormP_F1 (*n* = 5)	ePE_F1 (*n* = 5)	ePE + apo_F1 (*n* = 5)	Effect of apo *p* value one‐way ANOVA
ICAM‐1 (pg/mL)	♂	8057 ± 250	8359 ± 901	9501 ± 766*	*p* = 0.34
	♀	6845 ± 445	7176 ± 875	6226 ± 218	*p* = 0.52
VCAM1 (pg/mL)	♂	8527 ± 1406	6462 ± 1300	7475 ± 1043	*p* = 0.53
	♀	6296 ± 1678	4727 ± 836	6998 ± 1077	*p* = 0.44
TNFα (pg/mL)	♂	6.68 ± 5.55	0.66 ± 0.69	53.51 ± 29.69	*p* = 0.06
	♀	21.12 ± 6.95	0.42 ± 0.42	68.97 ± 41.61	*p* = 0.17
IL‐17A (pg/mL)	♂	3.85 ± 0.51	4.62 ± 0.73	4.21 ± 0.60	*p* = 0.69
	♀	4.14 ± 0.42	4.01 ± 0.62	5.93 ± 0.64	*p* = 0.06
IL‐1β (pg/mL)	♂	12.53 ± 18.56	0.99 ± 0.99	167.41 ± 90.61	*p* = 0.051
	♀	43.13 ± 20.53	0.00 ± 0.00	120.71 ± 36.82**	*p* = 0.01
IL‐1α (pg/mL)	♂	9.37 ± 9.09	12.65 ± 13.41	11.42 ± 12.77	*p* = 0.97
	♀	3.91 ± 3.89	12.07 ± 7.28	9.17 ± 5.51	*p* = 0.43

* *p* < 0.05 Results from Luminex analysis in plasma samples from NormPreg, ePE and ePE + apo adult offspring. ePE + apo_F1 males had increased levels of ICAM‐1 compared to ePE + apo_F1 females (*p < 0.05 vs. female by unpaired *t*‐test, Welch's correction).

** *p* < 0.01 ePE + apo_F1 females had increased levels of IL‐1β compared to ePE_F1 females (**p < 0.01 vs. ePE_F1 by one‐way ANOVA with Tukey's multiple comparisons).

## 4 Discussion

Maternal PE represents a developmental insult for offspring with lifelong consequences including cognitive dysfunction from childhood through adulthood [43, 44]. Importantly, offspring from preeclamptic pregnancies have been shown to have alterations in neuroanatomy and functional brain connectivity [45, 46], indicating long‐lasting effects on the offspring brain. Further, male offspring experience greater cognitive impairment than females, supporting that the response to the developmental insult of PE is sex‐related [13]. In addition to brain connectivity, offspring from PE mothers have been shown to have smaller diameter of cerebral vessels, demonstrating a long‐lasting effect in the cerebral vasculature [47]. In the current study, we found that juvenile male ePE_F1 had smaller body weights compared to NormPreg_F1 males. In addition, ePE_F1 males had increased BBB permeability associated with increased myogenic tone. Maternal treatment with a non‐selective antioxidant during gestation prevented these alterations induced by ePE exposure in male offspring. These adverse effects appeared sex‐specific as there was little effect of ePE pregnancy on females. Although the mechanisms by which PE pregnancy affects brain development are complex and multifactorial, increased reactive oxygen species (ROS) production in PE women, especially the placenta, may have a role that was confirmed by the current study [48]. In addition, the embryonic brain is highly susceptible to injury by ROS due to its low antioxidant capacity [49], potentially contributing to the enduring consequences observed on brain and cerebrovascular health.

Other animal studies have demonstrated the adverse effect of exposure to ePE on offspring brains [29, 30, 31, 50]. In agreement with Zheng et al. [51], we found exposure to ePE had a greater impact on the cerebrovasculature from male compared to female offspring.

The sexual dimorphism observed may be due to estrogen which exerts protective effects against oxidative stress conferring a protective phenotype to females [52]. The results from this study cannot determine the underlying mechanism that causes this sexual dimorphic response to the adverse in‐utero environment. Thus, future work is needed to address this.

Here, we demonstrated that exposure to ePE significantly increased BBB permeability in male offspring that was prevented by maternal treatment with apocynin, suggesting that increased BBB permeability in adult offspring was caused by increased ROS expression in‐utero. ROS can damage the BBB by promoting the expression of matrix metalloproteinases proteins, which increase inflammatory mediators [35]. This process leads to BBB disruption through the degradation of tight junction and basement membrane proteins [35]. The compromised BBB activates microglia, which in turn, release TNF‐α, further promoting neuroinflammation [53]. This cascade can impair hippocampal network dynamics and ultimately result in memory dysfunction [54]. Thus, this finding provides evidence that the adverse in‐utero environment in ePE dams had a persistent effect on offspring cerebrovasculature. Although we did not find differences in the inflammatory markers analyzed, evidence of systemic inflammation has been observed at a younger age [17] in preeclamptic offspring. Thus, ePE may induce a transient state of inflammation, not persistent in adulthood, which may affect cerebrovascular development.

We also found that exposure to ePE in‐utero caused PCAs to have modestly increased myogenic tone in male offspring. The PCA diameter of male ePE_F1 was only slightly reduced and not statistically significant; however, it is important to emphasize its hemodynamic relevance as blood flow is inversely proportional to the fourth power of the vessel radius [55]. Therefore, the small reduction in diameter of the PCAs could have a large effect on increasing vascular resistance and may reduce cerebral blood flow to the hippocampus. Hippocampal hypoperfusion is considered a primary predictor of cognitive decline [56]. We also found that PCAs from male ePE_F1 were slightly stiffer compared to those from male NormPreg_F1. These structural findings are similar to our previous study that found middle cerebral arteries from ePE offspring did not enlarge over maturation [17], whether this persisted into adulthood or was sex‐specific was not determined in that study. In another study in mice, it was shown that exposure to maternal hypertension induced profound vascular remodeling of the carotid arteries in male adult offspring due to the aberrant transmission of the methylation pattern of N6‐methyladenosine [51].

Together with the current study, this evidence supports the concept that exposure to PE induces permanent vascular changes that may underline pathological phenotypes later in life.

We investigated the reactivity of PCAs to mediators of NVC, including inward potassium rectifier channel [57] and nitric oxide [58], as they are an important link between vascular function and cognition. Although these findings did not support the hypothesis of impairment to reactivity of mediators of NVC, it is important to consider that changes in other mediators that are involved in neuronal activity and synaptic transmission, either from neurons or astrocytes, that we did not measure, may be impaired [23].

We found that juvenile male ePE_F1 had reduced body weights compared to male NormPreg_F1. The body weight for the age range studied is equivalent to young adult in humans. Indeed, studies in humans have demonstrated that reduced weight during early childhood is associated with increased risk of cerebrovascular disease during adulthood [56]. Hence, it is possible that a reduction in body weight during this period affected the structure and function of the offspring cerebral circulation. Small for gestational age at birth is a well‐established risk factor for long‐term cognitive deficits, and is often a consequence of fetal growth restriction, which can occur in PE [3, 59]. Although we did not measure the weight at birth, it is possible that the HC diet caused reduced fetal weight in pregnant dams. Thus, if present, it is essential to distinguish whether the cognitive effects in offspring are primarily due to the maternal preeclamptic environment, the consequences of restricted weight at birth, or a combination of both.

In the present study, maternal treatment with apocynin prevented the cerebrovascular alterations induced by ePE in male offspring. Apocynin, due to its broad antioxidant properties, may prevent maternal oxidative stress during ePE, thus creating a more favorable in‐utero environment for fetal development. In a rat model of PE, apocynin has been shown to reduce the antiangiogenic molecule soluble fms‐like tyrosine kinase‐1, while increasing placental growth factor levels, which are critical for vascular homeostasis during pregnancy [37]. In addition, apocynin has been shown to attenuate both systemic and placental inflammation by inhibiting the placental TLR4/NF‐kB signaling pathway [37]. However, as a lipophilic molecule, apocynin could cross the placenta and may directly target the fetal tissues, including the brain, preventing the alterations observed in ePE. Also, a concurrent action on both in‐utero environment and fetal tissue could not be ruled out. Therefore, further studies are required to elucidate the mechanisms by which apocynin contributes to the preservation of the long‐term cerebrovascular health of the offspring.

A limitation of the study is the age range of our experimental groups, due to the number of animals needed which included biological replicates and the time required to perform the experiments. However, all animals in the study were 3–7 months old, and considering that the rat lifespan is ~30 months, all animals are considered young adults. Although hormonal variations could potentially influence offspring phenotype across the 3–7‐month age range, this period is characterized by relative hormonal stability in both male and female rats [60]. Thus, potential fluctuations in sex hormone levels are minimal and unlikely to significantly confound the observed outcomes. It is noteworthy that we observed sex‐specific differences among pups exposed to the same in‐utero environment. These finding suggest that sex‐related outcomes may emerge even in the absence of broader litter‐based variation, hence providing preliminary evidence into sex‐specific programming effects on cerebrovasculature during pre‐natal life.

## 5 Perspectives

The current study found that ePE had long‐lasting, sex‐specific consequences on adult offspring cerebrovasculture, demonstrating that the altered intrauterine environment during ePE represents a risk factor for offspring well‐being through the entire lifespan. Remarkably, maternal treatment during gestation prevented the long‐term effects on offspring vascular health. Overall, our findings provide insight into the progressive effects of PE on PCAs alterations in adult offspring and the possibility of addressing maternal treatment to improve offspring well‐being later in life.

## Funding

This project was supported by the National Institutes of Health Grant #P20GM135007‐04S1 Centers of Biomedical Research Excellence (COBRE) Supplement from the National Institute of General Medicine and the National Institute of Neurological Disorders and Stroke grant 2R01NS093289‐10. We appreciate the support and guidance of investigators from the Molecular Epidemiology and Biostatistics Core of the Vermont Center for Cardiovascular and Brain Health. Funding was provided by P20 GM135007 from the National Institute of General Medical Sciences. We also gratefully acknowledge the support of the Cardiovascular Research Institute of Vermont and the Totman Medical Research Trust.

## Ethics Statement

The animal study was reviewed and approved by the University of Vermont IACUC.

## Conflicts of Interest

The authors declare no conflicts of interest.

## Supporting information


**Figure S1:** Effect of ePE and maternal apocynin treatment on offspring PCA flux. (A) PCA volume flux comparison between male offspring from NormPreg, ePE and ePE + apo dams. Maternal ePE significantly increased the volume of water filtered through the vessel wall in ePE_F1 males compared to NormPreg_F1 males after 20 min (^†^ *p* < 0.05 vs. NormPreg_F1 by two‐way ANOVA Holm‐Šídák's post hoc), that was prevented by apocynin (^^*p* < 0.01, ^*p* < 0.05 vs. ePE + apo_F1 by two‐way ANOVA Holm‐Šídák's post hoc). (B) PCA flux comparison between female offspring from NormPreg, ePE and ePE + apo dams. The volume of water filtered through the vessel was similar between NormPreg_F1, ePE_F1, and ePE + apo_F1 females. Males and females from NormPreg_F1 (C) and ePE_F1 (D), had no difference in the volume flux through the vessel wall. (E) Only PCAs from male ePE + apo_F1 had reduced volume filtered through the vessel wall compared to ePE + apo_F1 females (*p < 0.05 vs. female Multiple Mann–Whitney *t*‐test).
**Figure S2:** Effect of ePE and maternal apocynin on PCA transvascular filtration (J_v_/S) of offspring. Graphs showing J_v_/S of PCAs in response to increased hydrostatic pressure in male (A) and female (B) offspring from all groups. J_v_/S was increased in PCAs from ePE_F1 vs. NormPreg_F1 males that was prevented by apocynin (^*p* < 0.05 vs. ePE + apo_F1 by multiple comparison Holm‐Šídák's post hoc). J_v_/S was similar in PCAs from all female groups of offspring (NormPreg_F1, ePE_F1 and ePE + apo_F1 females). There were no sex‐differences in NormPreg_F1 (C) or ePE_F1 (D) males versus females in transvascular filtration. (E) Only PCAs from male ePE + apo_F1 had reduced J_v_/S compared to ePE + apo_F1 females (*p < 0.05 vs. female by unpaired multiple *t*‐test, Mann Whitney correction).
**Figure S3:** Effect of ePE and maternal apocynin on PCA structural remodeling of offspring. (A) Graph showing the percent distensibility of PCA of male offspring across the intravascular pressure range 5–200 mmHg. There was no difference in distensibility between NormPreg_F1, ePE_F1 and ePE + apo_F1 males. (B) Graph showing cross‐sectional area in PCAs from male offspring. There was no difference between PCAs from NormPreg_F1, ePE_F1 and ePE + apo_F1 males. (C) Graph of stress–strain curves for PCAs from male offspring. Vascular stiffness was not different between NormPreg_F1, ePE_F1, ePE + apo_F1 males. Graphs showing percent of distensibility (D), cross‐sectional area (E) and wall stress–strain curves (F) in PCAs from all groups of female offspring. There were no significant differences between groups.
**Figure S4:** Effect of ePE and maternal apocynin on PCA passive diameters and cross‐sectional area of offspring–effect of sex. (A–C) Graphs comparing male vs. female PCA passive inner diameters in offspring from all groups. Only ePE + apo_F1 males were larger compared to ePE + apo_F1 females at higher pressure (*p < 0.05 vs. female by unpaired *t*‐test, Welch's correction). There was no sex differences found in the other groups. (D–F) Graphs comparing male versus female PCA cross‐sectional area in all groups of offspring. Only ePE + apo_F1 males had increased cross‐sectional area versus females (*p < 0.05 vs. female by un‐paired *t*‐test, Welch's correction). There was no sex differences found in the other groups.
**Figure S5:** Effect of ePE and maternal apocynin on measures of PCA stiffness in offspring—effect of sex. (A–C) Graphs comparing male versus female PCA wall stress–strain curves. Only NormPreg_F1 females had increased stiffness compared to male (leftward shift). No sex difference found in the other groups. (D–F) Graphs comparing male versus female PCA distensibility in all groups of offspring. Only NormPreg_F1 males had increased distensibility versus females (*p < 0.05 vs. female by unpaired *t*‐test, Welch's correction). There was no sex differences found in the other groups.
**Figure S6:** PCAs constriction to L‐NAME in offspring from ePE dams with/without apocynin treatment. PCAs from all groups constricted to NOS inhibition with L‐NAME. The percent of constriction to L‐NAME was similar between males and females from NormPreg_F1, ePE_F1 and ePE + apo_F1.
**Figure S7:** Effect of ePE and maternal apocynin on PCA sensitivity to sodium nitroprusside (SNP) in offspring. (A, B) Graph showing sensitivity to increasing concentrations of SNP in male and female offspring. Male and female NormPreg_F1, ePE_F1 and ePE + apo_F1 dilated similarly in response to SNP. (C) Table of EC50 values to SNP in PCAs from all groups of offspring. There was no difference in the EC50 to SNP between male and female NormPreg_F1, ePE_F1, and ePE + apo_F1 (One‐way ANOVA, Holm‐Šídák's post hoc for group effects; unpaired *t*‐test, Welch's correction for sex differences).

## Data Availability

The datasets used and/or analyzed during the current study are available from the corresponding author upon reasonable request.
