# Supplementary material for: Maternal Apocynin During Experimental Preeclampsia Prevents BBB Permeability and Increased Vascular Tone of Cerebral Arteries in Male but Not Female Rat Adult Offspring
Source: Microcirculation. 2025 Dec 17;33(1):e70044. doi: 10.1111/micc.70044 (PMC12710456; doi:10.1111/micc.70044)
Supplement: Supplementary file 1 — Figure S1: Effect of ePE and maternal apocynin treatment on offspring PCA flux. (A) PCA volume flux comparison between male offspring from NormPreg, ePE and ePE + apo dams. Maternal ePE significantly increased the volume of water filtered through the vessel wall in ePE_F1 males compared to NormPreg_F1 males after 20 min († p < 0.05 vs. NormPreg_F1 by two‐way ANOVA Holm‐Šídák's post hoc), that was prevented by apocynin (^^p < 0.01, ^p < 0.05 vs. ePE + apo_F1 by two‐way ANOVA Holm‐Šídák's post hoc). (B) PCA flux comparison between female offspring from NormPreg, ePE and ePE + apo dams. The volume of water filtered through the vessel was similar between NormPreg_F1, ePE_F1, and ePE + apo_F1 females. Males and females from NormPreg_F1 (C) and ePE_F1 (D), had no difference in the volume flux through the vessel wall. (E) Only PCAs from male ePE + apo_F1 had reduced volume filtered through the vessel wall compared to ePE + apo_F1 females (*p < 0.05 vs. female Multiple Mann–Whitney t‐test). Figure S2: Effect of ePE and maternal apocynin on PCA transvascular filtration (Jv/S) of offspring. Graphs showing Jv/S of PCAs in response to increased hydrostatic pressure in male (A) and female (B) offspring from all groups. Jv/S was increased in PCAs from ePE_F1 vs. NormPreg_F1 males that was prevented by apocynin (^p < 0.05 vs. ePE + apo_F1 by multiple comparison Holm‐Šídák's post hoc). Jv/S was similar in PCAs from all female groups of offspring (NormPreg_F1, ePE_F1 and ePE + apo_F1 females). There were no sex‐differences in NormPreg_F1 (C) or ePE_F1 (D) males versus females in transvascular filtration. (E) Only PCAs from male ePE + apo_F1 had reduced Jv/S compared to ePE + apo_F1 females (*p < 0.05 vs. female by unpaired multiple t‐test, Mann Whitney correction). Figure S3: Effect of ePE and maternal apocynin on PCA structural remodeling of offspring. (A) Graph showing the percent distensibility of PCA of male offspring across the intravascular pressure range 5–200 mmHg. There was [file MICC-33-e70044-s001.docx]

**Supporting Information**

**Maternal Apocynin during Experimental Preeclampsia Prevents BBB Permeability and Increased Vascular Tone of Cerebral Arteries in Male but not Female Rat Adult Offspring**

Milena Esposito^a,b^, Sarah M. Tremble^a^, Marilyn J. Cipolla^a,c,d,^

^a^Department of Neurological Sciences, University of Vermont Larner College of Medicine, Burlington, VT, USA

^b^Department of Biology, Ecology and Earth Science, University of Calabria, Arcavacata di Rende, CS, Italy

^c^Department of Obstetrics, Gynecology and Reproductive Sciences, University of Vermont Larner College of Medicine, Burlington, VT, USA

^d^Department of Pharmacology, University of Vermont Larner College of Medicine, Burlington, VT, USA

**Corresponding Author:**

Marilyn J. Cipolla, PhD

University of Vermont Department of Neurological Sciences

149 Beaumont Ave.

Burlington, VT 05405

Phone: 802-656-9714

Email: marilyn.cipolla@med.uvm.edu

## *Data Calculations*

Hydraulic conductivity was calculated by the equation:

1. *L*_p_ = 𝐽𝑣/((𝑆×∆𝑃))

where *Jv*/S is the transvascular filtration

Transvascular filtration for surface area (*Jv/S*) was calculated by the equation:

1. *Jv/S* = ∆V/(∆t×S)

where Δ𝑉=volume flux, Δ𝑡=time interval and 𝑆=𝜋×𝐿×𝐷 (surface area).

Percent tone was calculated by the equation:

1. %tone = [(Ø_passive_- Ø_active_)/ Ø_passive_)×100%]

where Ø_passive_ is the diameter in zero-Ca^2+^ aCSF and Ø_active_ is the diameter when the vessels have tone.

Percent distensibility was calculated using the equation:

1. %distensibility = [(Ø_passive_– Ø_5_)/ Ø_5_]x100%

where Ø_5_ is the inner diameter of the fully relaxed vessel at an intravascular pressure of 5 mmHg.

Percent change of lumen diameter in response to different concentrations of K^+^ were calculated using the equation:

1. %change = [(Ø_dose_– Ø_baseline_)/(Ø_baseline_)]x100%

Wall tension was calculated across the pressure range 5–200 mmHg by converting pressure (mmHg) into dynes/cm^2^ × (Ø_inner_/2)

Wall stress was calculated at each pressure by the equation:

(6) wall stress = [(wall tension/wall thickness)]

Wall strain was calculated by the equation

1. wall strain = [(Ø_passive_ – Ø_mmHg_)/Ø_mmHg_]

where Ø_mmHg_ is the passive diameter at each pressure.

Outer diameter (Ø_outer_) was calculated at each pressure by the equation:

1. Ø_outer_ = [(Ø_inner_ +2WT)]

where Ø_inner_ is the inner diameter of the vessel fully relaxed and WT is the measured wall thickness.

Cross-sectional area (CSA) was calculated by the equation:

1. CSA = [π(Ø_outer_/2)2—π(Ø_inner_/2)2] at each intravascular pressure.

Percent sensitivity to SNP was calculated by the equation:

1. % sensitivity = [(Ø_dose_– Ø_baseline_)/(Ø_ID max dose_– Ø_baseline_)]x100%

where Ø_dose_ is the diameter of the vessel after treatment with a specific concentration of drug, Ø_baseline_ is the starting diameter before any drug treatment and Ø_ID_ _max dose_ is the diameter of the vessel at the highest concentration of the drug.

The EC_50_ for SNP (i.e., the amount of drug necessary to dilate the arteries 50% of maximum) was calculated for each artery by first plotting the concentration-response curves (sensitivity) on a logarithmic scale and extrapolating the value from a best- fit line between 20% and 80% dilation.

The percent constriction to L-NAME was calculated by the equation:

1. %constriction = [(Ø_baseline_-Ø_drug_)/ Ø_baseline_) × 100%],

where Ø_baseline_ is diameter before adding L-NAME and Ø_drug_ is diameter in presence of L-NAME.


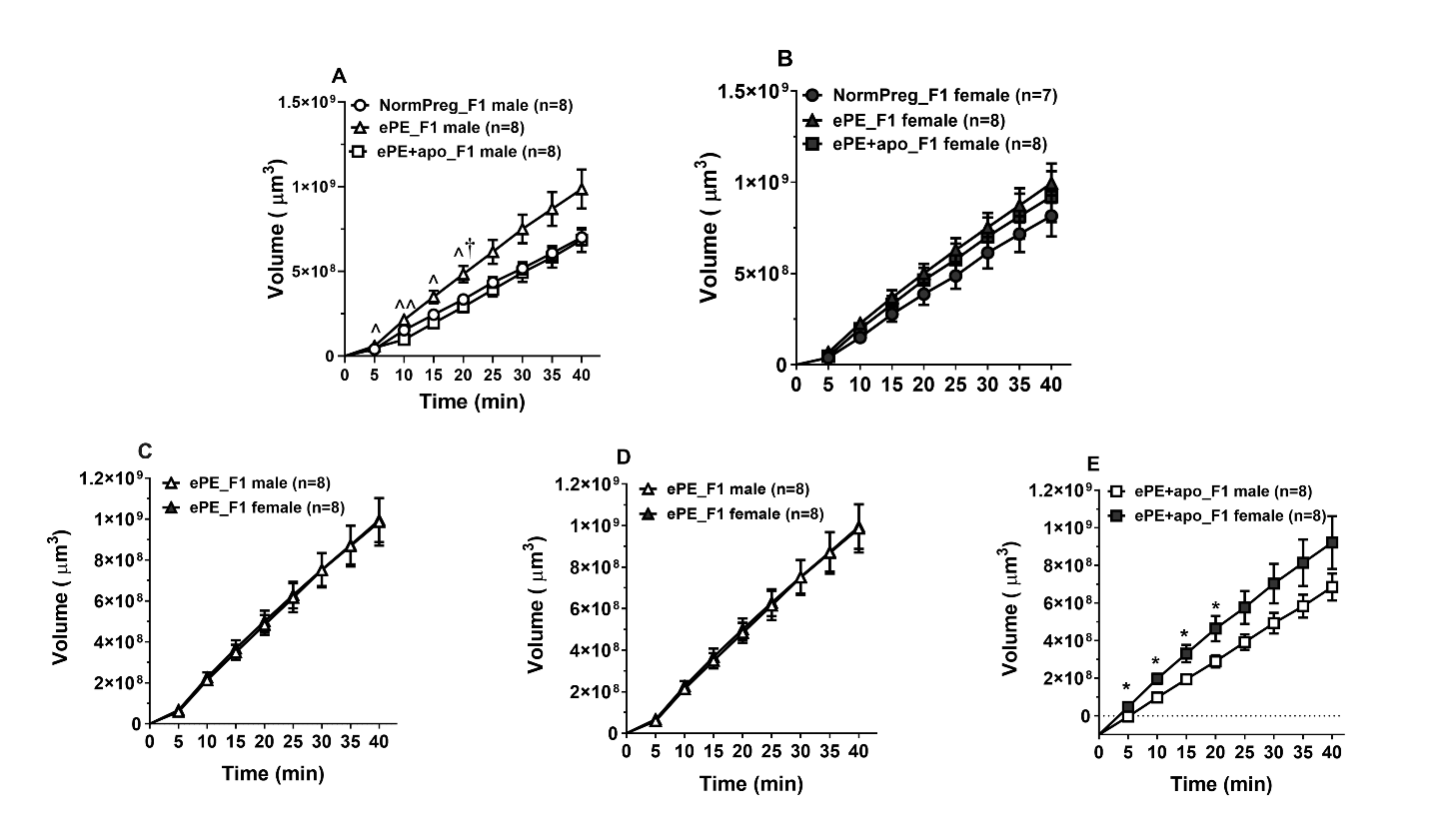


*Figure S1.* Effect of ePE and maternal apocynin treatment on offspring PCA flux*.* (A) PCA volume flux comparison between male offspring from NormPreg, ePE and ePE+apo dams. Maternal ePE significantly increased the volume of water filtered through the vessel wall in ePE_F1 males compared to NormPreg_F1 males after 20 minutes (^†^p<0.05 vs. NormPreg_F1 by two-way ANOVA Holm-Šídák’s post hoc), that was prevented by apocynin (^^p<0.01, ^p<0.05 vs. ePE+apo_F1 by two-way ANOVA Holm-Šídák’s post hoc). (B) PCA flux comparison between female offspring from NormPreg, ePE and ePE+apo dams. The volume of water filtered through the vessel was similar between NormPreg_F1, ePE_F1 and ePE+apo_F1 females. Males and females from NormPreg_F1 (C) and ePE_F1 (D), had no difference in the volume flux through the vessel wall. (E) Only PCAs from male ePE+apo_F1 had reduced volume filtered through the vessel wall compared to ePE+apo_F1 females (*p<0.05 vs. female Multiple Mann-Whitney t-test).


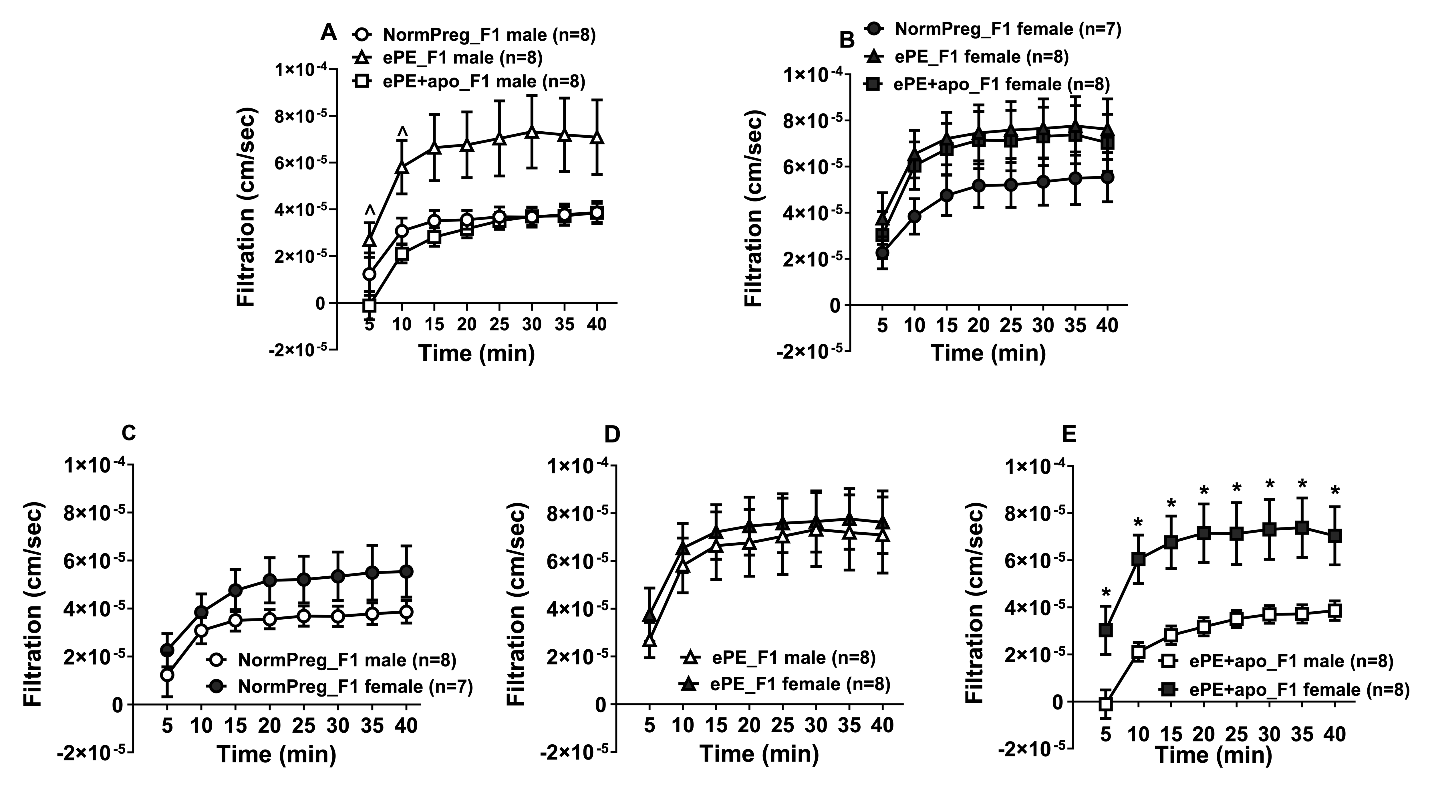


*Figure S2*: Effect of ePE and maternal apocynin on PCA transvascular filtration (*Jv/S*) of offspring. Graphs showing *Jv/S* of PCAs in response to increased hydrostatic pressure in male (A) and female (B) offspring from all groups. Jv/S was increased in PCAs from ePE_F1 vs. NormPreg_F1 males that was prevented by apocynin (^p<0.05 vs. ePE+apo_F1 by multiple comparison Holm-Šídák’s post hoc). Jv/S was similar in PCAs from all female groups of offspring (NormPreg_F1, ePE_F1 and ePE+apo_F1 females). There were no sex-differences in NormPreg_F1 (C) or ePE_F1 (D) males vs females in transvascular filtration. (E) Only PCAs from male ePE+apo_F1 had reduced Jv/S compared to ePE+apo_F1 females (*p<0.05 vs female by unpaired multiple t-test, Mann Whitney correction).

*
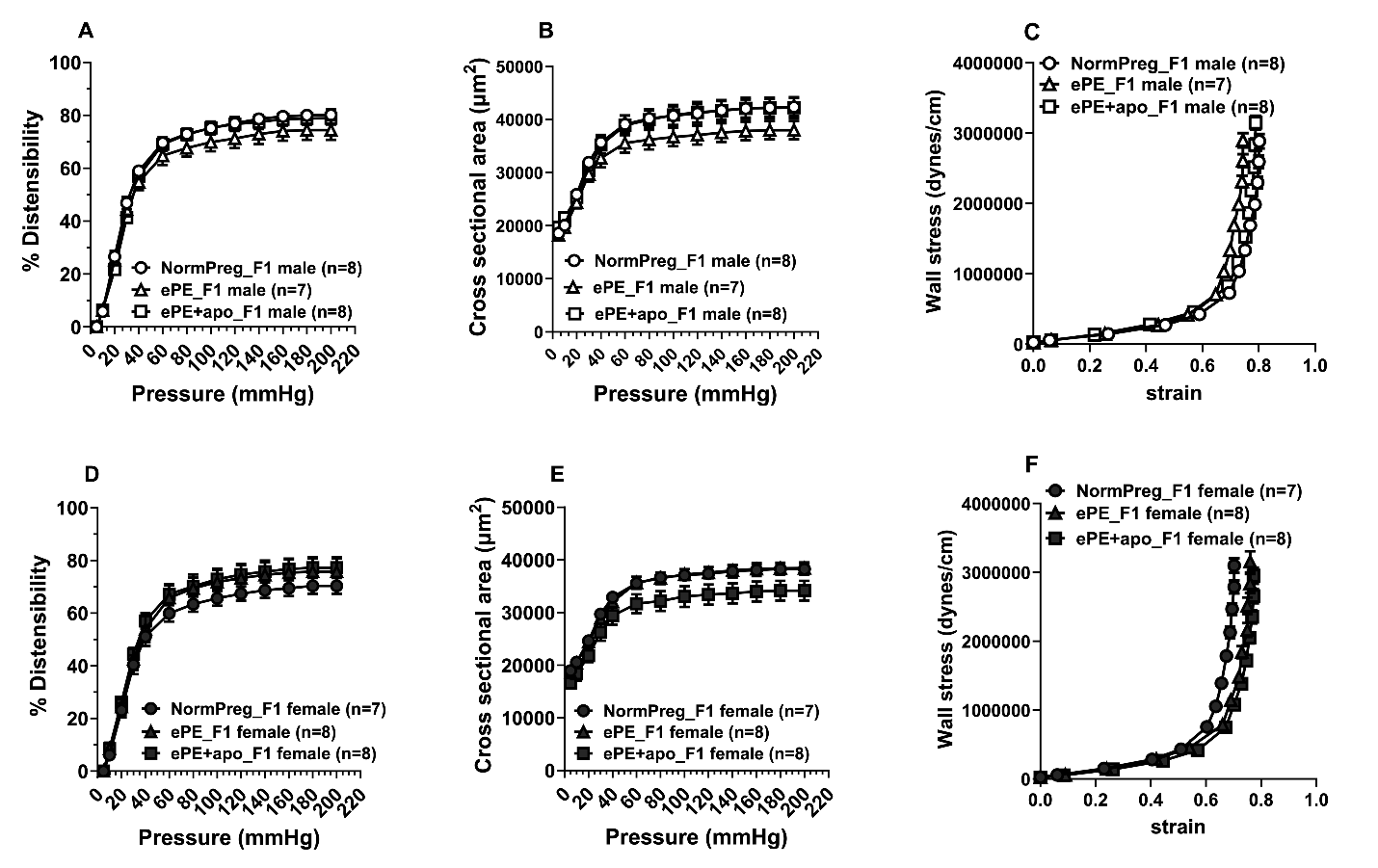
*

*Figure S3*: Effect of ePE and maternal apocynin on PCA structural remodeling of offspring. (A) Graph showing the percent distensibility of PCA of male offspring across the intravascular pressure range 5-200 mmHg. There was no difference in distensibility between NormPreg_F1, ePE_F1 and ePE+apo_F1 males. (B) Graph showing cross-sectional area in PCAs from male offspring. There was no difference between PCAs from NormPreg_F1, ePE_F1 and ePE+apo_F1 males. (C) Graph of stress-strain curves for PCAs from male offspring. Vascular stiffness was not different between NormPreg_F1, ePE_F1, ePE+apo_F1 males. Graphs showing percent of distensibility (D), cross-sectional area (E) and wall stress-strain curves (F) in PCAs from all groups of female offspring. There were no significant differences between groups.

*
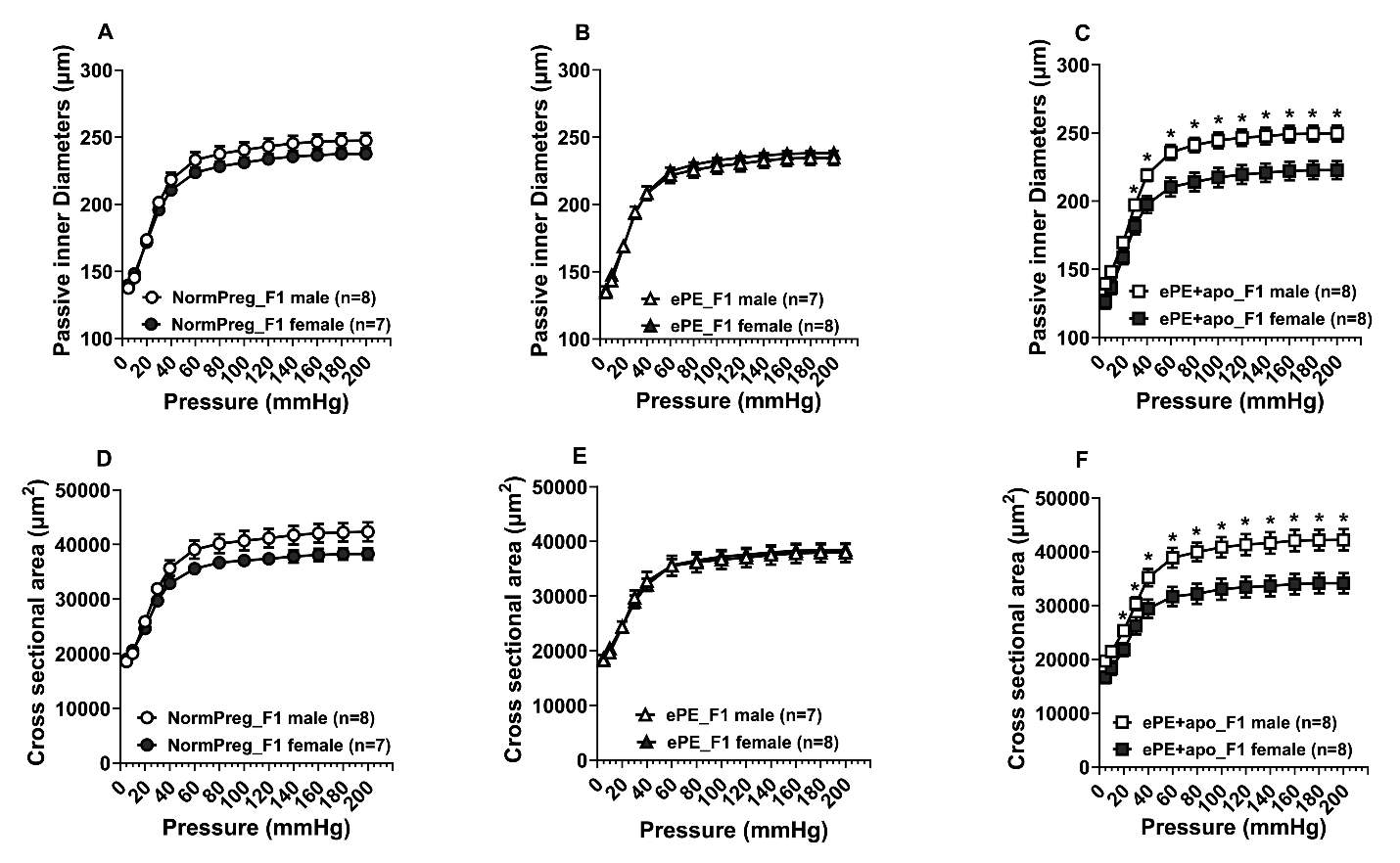
Figure S4*: Effect of ePE and maternal apocynin on PCA passive diameters and cross-sectional area of offspring–effect of sex. (A-C) Graphs comparing male vs. female PCA passive inner diameters in offspring from all groups. Only ePE+apo_F1 males were larger compared to ePE+apo_F1 females at higher pressure (*p<0.05 vs female by un-paired t-test, Welch’s correction). There was no sex differences found in the other groups. (D-F) Graphs comparing male vs. female PCA cross-sectional area in all groups of offspring. Only ePE+apo_F1 males had increased cross-sectional area vs. females (*p<0.05 vs. female by un-paired t-test, Welch’s correction). There was no sex differences found in the other groups.


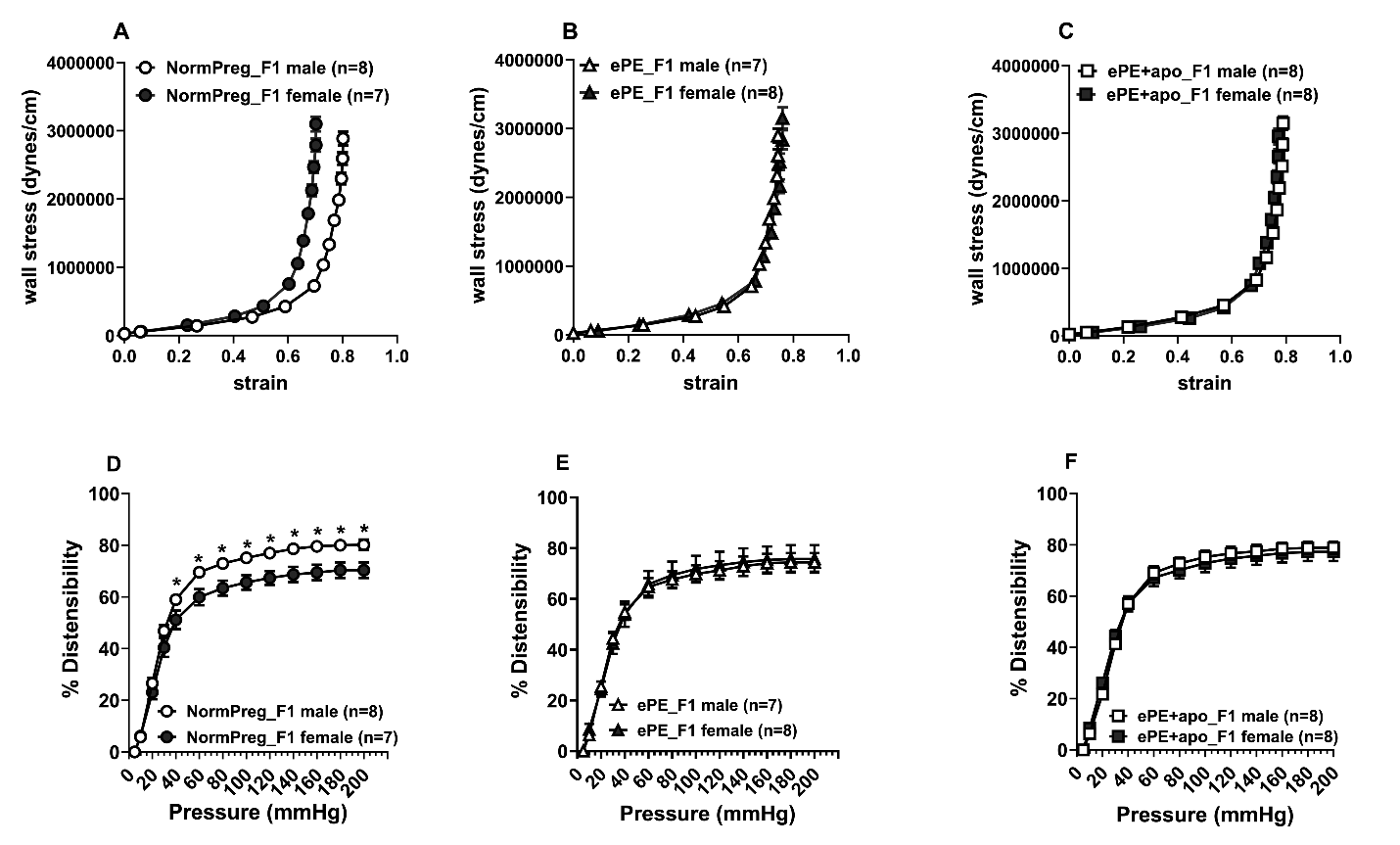


*Figure S5*: Effect of ePE and maternal apocynin on measures of PCA stiffness in offspring – effect of sex. (A-C) Graphs comparing male vs. female PCA wall stress-strain curves. Only NormPreg_F1 females had increased stiffness compared to male (leftward shift). No sex difference found in the other groups. (D-F) Graphs comparing male vs. female PCA distensibility in all groups of offspring. Only NormPreg_F1 males had increased distensibility vs. females (*p<0.05 vs. female by un-paired t-test, Welch’s correction). There was no sex differences found in the other groups.

*
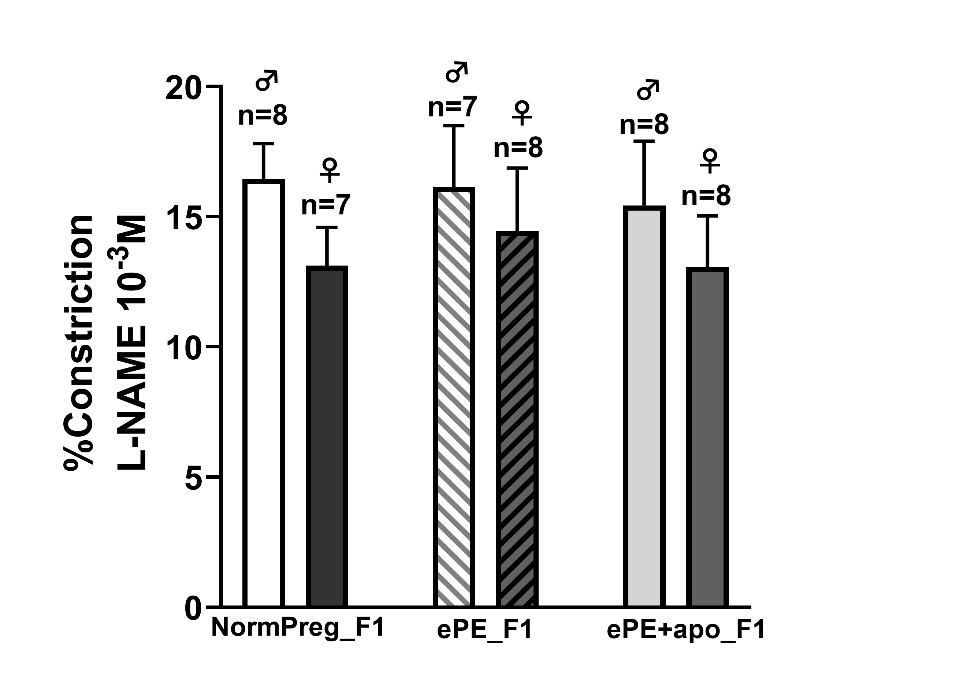
*

*Figure S6*: PCAs constriction to L-NAME in offspring from ePE dams with/without apocynin treatment. PCAs from all groups constricted to NOS inhibition with L-NAME. The percent of constriction to L-NAME was similar between males and females from NormPreg_F1, ePE_F1 and ePE+apo_F1.


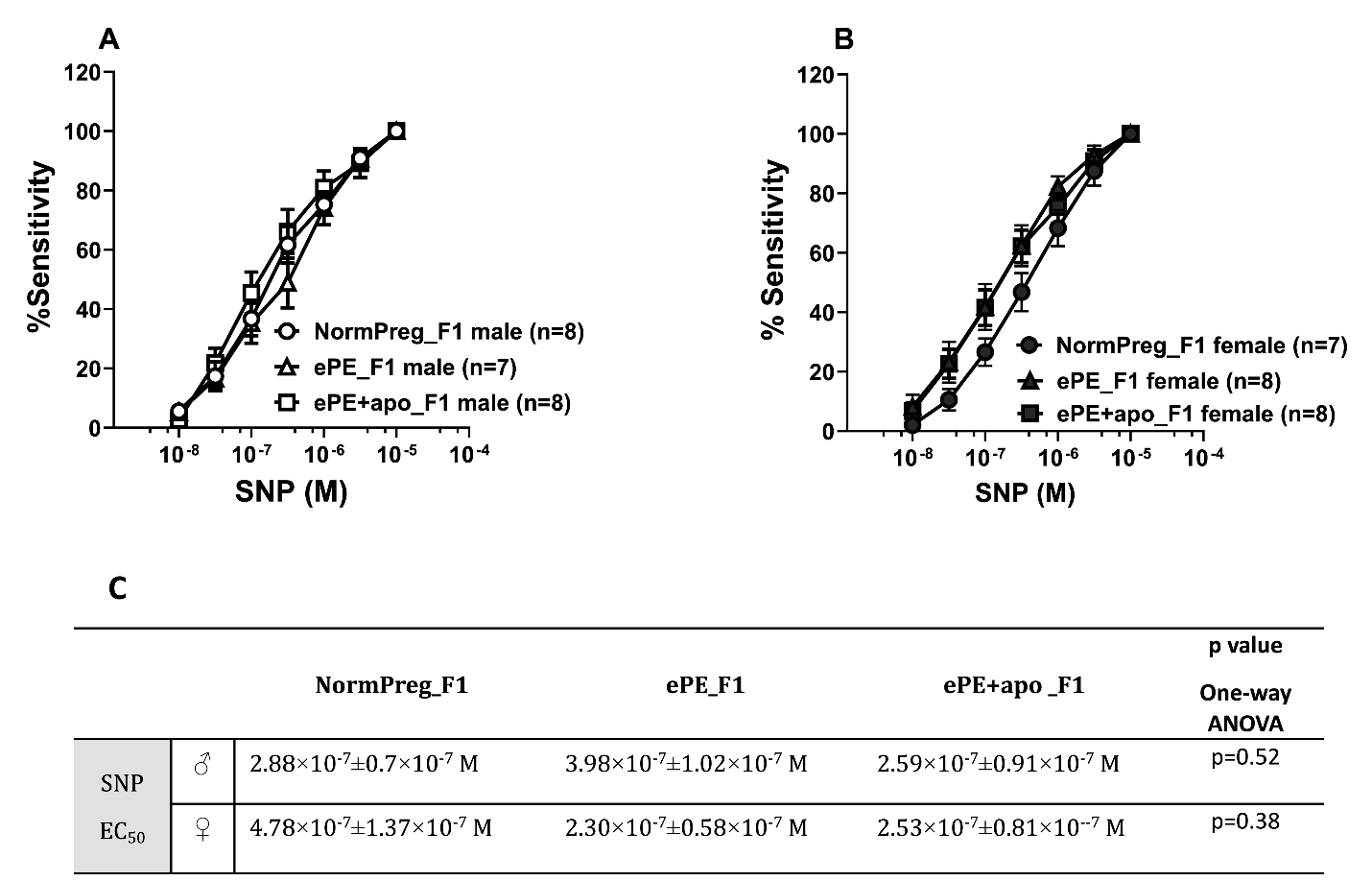


*Figure S7:* Effect of ePE and maternal apocynin on PCA sensitivity to sodium nitroprusside (SNP) in offspring. (A, B) Graph showing sensitivity to increasing concentrations of SNP in male and female offspring. Male and female NormPreg_F1, ePE_F1 and ePE+apo_F1 dilated similarly in response to SNP. (C) Table of EC_50_ values to SNP in PCAs from all groups of offspring. There was no difference in the EC_50_ to SNP between male and female NormPreg_F1, ePE_F1 and ePE+apo_F1 (One-way ANOVA, Holm-Šídák’s post-hoc for group effects; un-paired t-test, Welch's correction for sex differences).
